# Transcriptional activator Cat8 is involved in regulation of xylose alcoholic fermentation in the thermotolerant yeast *Ogataea (Hansenula) polymorpha*

**DOI:** 10.1186/s12934-017-0652-6

**Published:** 2017-02-28

**Authors:** Justyna Ruchala, Olena O. Kurylenko, Nitnipa Soontorngun, Kostyantyn V. Dmytruk, Andriy A. Sibirny

**Affiliations:** 1 Department of Biotechnology and Microbiology, University of Rzeszow, Zelwerowicza 4, 35-601 Rzeszow, Poland; 2 Department of Molecular Genetics and Biotechnology, Institute of Cell Biology, Drahomanov Str., 14/16, Lviv, 79005 Ukraine; 3 King Mongkut Technical University, Thonbury, Thailand

**Keywords:** Transcriptional activator, Xylose, High-temperature alcoholic fermentation, Yeast, *Ogataea (Hansenula) polymorpha*

## Abstract

**Background:**

Efficient xylose alcoholic fermentation is one of the key to a successful lignocellulosic ethanol production. However, regulation of this process in the native xylose-fermenting yeasts is poorly understood. In this work, we paid attention to the transcriptional factor Cat8 and its possible role in xylose alcoholic fermentation in *Ogataea (Hansenula) polymorpha.* In *Saccharomyces cerevisiae,* organism, which does not metabolize xylose, gene *CAT8* encodes a Zn-cluster transcriptional activator necessary for expression of genes involved in gluconeogenesis, respiration, glyoxylic cycle and ethanol utilization. Xylose is a carbon source that could be fermented to ethanol and simultaneously could be used in gluconeogenesis for hexose synthesis. This potentially suggests involvement of *CAT8* in xylose metabolism.

**Results:**

Here, the role of *CAT8* homolog in the natural xylose-fermenting thermotolerant yeast *O. polymorpha* was characterized. The *CAT8* ortholog was identified in *O. polymorpha* genome and deleted both in the wild-type strain and in advanced ethanol producer from xylose. Constructed *cat8*Δ strain isolated from wild strain showed diminished growth on glycerol, ethanol and xylose as well as diminished respiration on the last substrate. At the same time,* cat8*Δ mutant isolated from the best available *O. polymorpha* ethanol producer showed only visible defect in growth on ethanol. *CAT8* deletant was characterized by activated transcription of genes *XYL3, DAS1* and *RPE1* and slight increase in the activity of several enzymes involved in xylose metabolism and alcoholic fermentation. Ethanol production from xylose in *cat8*Δ mutants in the background of wild-type strain and the best available ethanol producer from xylose increased for 50 and 30%, respectively. The maximal titer of ethanol during xylose fermentation was 12.5 g ethanol/L at 45 °C. Deletion of *CAT8* did not change ethanol production from glucose. Gene *CAT8* was also overexpressed under control of the strong constitutive promoter *GAP* of glyceraldehyde-3-phosphate dehydrogenase. Corresponding strains showed drop in ethanol production in xylose medium whereas glucose alcoholic fermentation remained unchanged. Available data suggest on specific role of Cat8 in xylose alcoholic fermentation.

**Conclusions:**

The *CAT8* gene is one of the first identified genes specifically involved in regulation of xylose alcoholic fermentation in the natural xylose-fermenting yeast *O. polymorpha*.

**Electronic supplementary material:**

The online version of this article (doi:10.1186/s12934-017-0652-6) contains supplementary material, which is available to authorized users.

## Background

Fermentation is the largest field of industrial biotechnology. In 2014, near 95 billion liters of ethanol were produced [1]. Currently, most of industrial ethanol is produced from starch and sucrose (1st generation ethanol), however, due to limited feedstock abundance, further increase in fuel ethanol production will depend on development of feasible technology of alcoholic fermentation from lignocellulose (2nd generation ethanol). One of the most important goals in the development of such technology is construction of strains capable of efficient fermentation of lignocellulosic pentoses, especially xylose, which constitutes about 30% of all sugars in lignocellulosic hydrolyzates [2, 3]. It would also be useful to carry out fermentation of xylose and other lignocellulosic sugars under elevated temperatures (around 50 °C), which would allow optimal activities of cellulases and hemicellulases necessary for the process known as Simultaneous Saccharification and Fermentation (SSF) [4]. In such a process, free sugars liberated by enzymatic hydrolysis do not exert product inhibition on hydrolyzing enzymes, since they are simultaneously converted to ethanol by thermotolerant microorganisms in the same vessel. Very few yeast organisms are capable of high-temperature alcoholic fermentation, namely *Kluyveromyces marxianus* [5] and *Ogataea (Hansenula) polymorpha* [6, 7]. Current work focuses on *O. polymorpha* which is the most thermotolerant yeast species known to date, with maximal growth and fermentation temperatures of 50 °C or even higher [8, 9]. It has been reported that *O. polymorpha* produces ethanol from glucose, cellobiose, glycerol and xylose at elevated temperatures [7, 10], however, ethanol yield and productivity from xylose by the wild-type strains is very low [11]. *O. polymorpha* can also produce ethanol directly from starch and xylan after expression of heterologous genes encoding corresponding hydrolytic enzymes [12]. Several methods of metabolic engineering, both original and those developed for other yeast species, were successfully used for improvements of ethanol synthesis from xylose in *O. polymorpha*. They include heterologous expression of bacterial xylose isomerases and overexpression of native xylulokinase [13] and, alternatively, overexpression of engineered xylose reductase with decreased affinity to NADPH as well as native xylitol dehydrogenase and xylulokinase [14] and overexpression of pyruvate decarboxylase in the strain unable to utilize ethanol as sole carbon source [15]. Combination of metabolic engineering (overexpression of engineered xylose reductase and native xylitol dehydrogenase and xylulokinase) with classical selection approaches (selection for strains unable to utilize ethanol as sole carbon source and resistant to glycolysis inhibitor 3-bromopyruvate), allowed isolation of strains that accumulate 15–20 times more ethanol from xylose relative to the wild-type strain, i.e. around 10 g ethanol/L at 45 °C [16]. While mutation(s) causing resistance to 3-bromopyruvate in the ethanol overproducing strain remain to be identified, we have recently mapped a corresponding mutation in the strain with the wild-type background and showed that it disrupted an autophagy-related gene *ATG13.* This mutation led to a 50% increase in ethanol production from xylose [17]; Dmytruk, Sibirny, in preparation]. Still, the achieved yield and productivity of ethanol synthesis from xylose are lower than that described for engineered *Saccharomyces cerevisiae* and several native xylose-fermenting yeasts (which however are mesophilic and therefore could not be useful for the SSF process). Further possible increase in ethanol synthesis by *O. polymorpha* from xylose is hampered due to absence of the knowledge on regulation of xylose metabolism and fermentation. Therefore, it is important to identify the corresponding genes and, depending on their functions, activate or repress them. Described functions of a transcription factor Cat8 (encoded by *CAT8* gene) in activating multiple metabolic processes in *S. cerevisiae*, mostly gluconeogenesis and ethanol utilization [18, 19], led us to hypothesize that it might also be involved in regulation of xylose metabolism in *O. polymorpha*. One of the reasons that just *CAT8* was selected among multiple genes coding for transcription factors involved in carbon metabolism [20] was that knock out of *CAT8* activated glucose alcoholic fermentation in *S. cerevisiae* [21] and non-conventional yeast *Pichia guilliermondii* [22]. Xylose is a unique carbon source as it could be fermented to ethanol, similarly to glucose, and simultaneously it has to be converted to glucose and other hexoses, mostly in pentose phosphate pathway though partial contribution of gluconeogenesis in hexose synthesis from xylose cannot be neglected. We hypothesized that for these reasons the mutants of *O. polymorpha* with knock out of the ortholog of *CAT8* gene will have impairments in xylose respiration and gluconeogenesis, so the flux of this sugar will be activated instead into fermentation direction.

Roles of *CAT8* gene in regulation of cell metabolism are quite well understood in *S. cerevisiae*. It encodes a Zn-cluster transcriptional activator necessary for expression of genes involved in gluconeogenesis, ethanol utilization and diauxic shift from fermentation to respiration [18, 19]. Strains with deletion of *CAT8* show defects in growth on ethanol, glycerol and other gluconeogenic substrates whereas disaccharides are utilized normally. Mechanistically, Cat8 exerts transcriptional activation of its target genes by binding to carbon source-responsive elements in their regulatory promoters [20, 23]. However, the limited data available on the functions of *CAT8* in non-*Saccharomyces* yeasts show differences in functions of the corresponding orthologs. Thus, *Kluyveromyces lactis* mutant defective in *CAT8* showed defects in ethanol utilization, whereas growth on glycerol was normal [24]. *cat8*Δ mutant of *Candida albicans* normally utilized all carbon substrates tested [25], whereas growth patterns of the mutant with knock out of *CAT8* in *Pichia guilliermondii* were not assayed at all [22]. Role of *CAT8* in regulation of xylose metabolism is poorly understood. Transcriptome analysis of the natural xylose-metabolizing yeast *O. polymorpha* did not find changes in *CAT8* expression between xylose- and glucose-containing media [26]. In recombinant *S. cerevisiae* capable of xylose metabolism, xylose caused only weak repression of *CAT8* relative to glucose suggesting xylose growing cells are in between totally repressed and derepressed state regarding catabolite repression [27].

To test our hypothesis on the role of Cat8 transcription factor in xylose fermentation, we have isolated *CAT8* knock-out mutants in *O. polymorpha* on either wild-type or ethanol overproducing (from xylose) strain [16]. We also overexpressed *CAT8*. In favor of our hypothesis, we found that strains with deletions of *CAT8*Δ accumulate more ethanol during xylose fermentation, while ethanol production from glucose was not changed. Mutant *O. polymorpha cat8* isolated from the advanced ethanol producer accumulated up to 12.5 g ethanol/L at 45 °C, which is the highest ethanol titer for high-temperature xylose fermentation. Inversely, strain of *O. polymorpha* with overexpression of *CAT8* accumulated less ethanol relative to the parental wild-type strain.

## Results

### Isolation and growth characteristics of *cat8*Δ mutants

We decided to delete the *O. polymorpha CAT8* ortholog in both wild-type strain and the best ethanol producer (BEP) from xylose [16] and to study the properties of the resulted deletants. In particular, we focused on growth patterns, respiration, activity of some enzymes, expression of selected genes and ethanol production in xylose and glucose media. Genome of *O. polymorpha* strain NCYC495 is sequenced and is publicly available [28]. It contains single ortholog of *S. cerevisiae* gene *CAT8,* which shows 31% identity and 53% similarity to *CAT8* gene of *S. cerevisiae*. To knock it out in *O. polymorpha*, a deletion cassette was constructed, which contained *natNT2* gene conferring resistance to nourseothricin as a selection marker, flanked with non-coding regions of the *CAT8* gene ortholog (see “Methods” section; Additional file 1A). Homologous recombination resulted in isolation of the *cat8*Δ strain. In total, near 1000 nourseothricin-resistant transformants were analyzed and 9 of them appeared to be *cat8*Δ mutants. Our attempts to isolate *cat8*Δ on the background of strain BEP were unsuccessful. In total, we analyzed near 2000 transformants and invariably without success. It is known that the selection marker has strong impact on the efficiency of homologous recombination [29]. Therefore, we decided to construct a deletion cassette using a selection marker gene *hphNT1*, conferring resistance to hygromycin (see “Methods” section; Additional file 1B). In this case, 10 *CAT8* knock out mutants were identified among 400 analyzed hygromycin-resistant transformants.

**Fig. 1:**
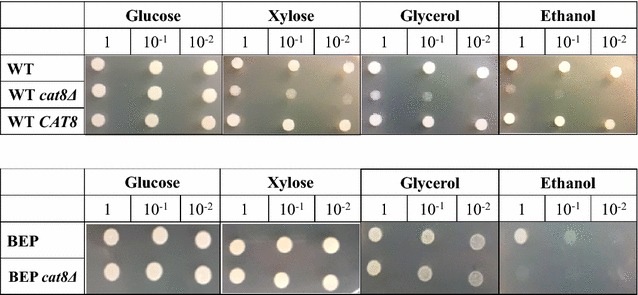
Growth of the strains with deletion (*cat8*∆) or overexpression of *CAT8* gene (CAT8*) on different carbon sources (glucose, xylose, glycerol, ethanol) as compared to the parental strains

The isolated *cat8*Δ mutants on the background of the wild-type and BEP strains were assayed for growth, biochemical and physiological characteristics. Growth of these mutants was analyzed in YNB solid and liquid media supplemented with different carbon sources and compared with that of the corresponding parental strain. It was found that isolated mutants normally grew in media with glucose, whereas growth of *cat8*Δ mutant isolated from wild-type strain on glycerol and ethanol was retarded but not totally abolished. Growth of BEP *cat8*Δ was very similar to that of BEP in glycerol containing medium, while BEP *cat8*Δ was unable to growth in ethanol, unlike to BEP (Additional file 2). It is remarkable that growth of *cat8*Δ strain isolated from the wild-type strain on xylose was also partially retarded, whereas no significant difference in growth on xylose was observed between BEP and BEP *cat8*Δ strains. However, the BEP strain much better grows on xylose relative to the wild-type strain apparently due to overexpression of genes *XYL1, XYL2* and *XYL3* involved in primary xylose metabolism [16] (Fig. 1; Additional file 2). It has to be pointed out that ethanol overproducing strain BEP poorly grows on ethanol [16], whereas its derivative BEP *cat8*Δ mutant did not grow on this substrate at all. We suggest that function of *CAT8* in *O. polymorpha* is similar to that in *S. cerevisiae* as corresponding deletants grow poorly on ethanol and glycerol.

### Isolation and growth characteristics of the strains with overexpression of *CAT8*

Transformants of *O. polymorpha* wild-type strain, which express *CAT8* under control of the strong constitutive *GAP* promoter of glyceraldehyde-3-phosphate dehydrogenase gene, were isolated (WT *CAT8*). Overexpression of *CAT8* was proved by qRT-PCR. It was found that indeed, the analyzed strain with *CAT8* gene under *GAP* promoter showed increase in *CAT8* expression for 2.65 times (Additional file 3). It was found that WT *CAT8* did not differ from the wild-type strain regarding growth on the tested substrates: glucose, xylose, glycerol and ethanol (Fig. 1).

### Respiration, enzymatic profiles and transcription of selected genes in the isolated mutants

More detailed physiological, biochemical and genetic analyses were carried out on constructed deletion mutants *cat8*Δ and BEP *cat8*Δ. To reveal the role of *CAT8* gene in the metabolism of *O. polymorpha*, cell respiration of *cat8*Δ cells in glucose- and xylose-containing media was studied. It was found that cells of both *cat8*Δ and BEP *cat8*Δ strains showed up to 40% decrease in respiration with xylose as a substrate. Respiration of *cat8*Δ but not that of BEP *cat8*Δ cells also was decreased using glucose as a substrate (Table 1). These data confirm our suggestion on the similar role of *CAT8* in *O. polymorpha* and *S. cerevisiae*. The observed small increase in glucose respiration of BEP *cat8*Δ cells apparently depends on unidentified mutations introduced in BEP strain during its selection [16]. In the following experiments, we analyzed specific activities of several enzymes involved in xylose metabolism and ethanol synthesis in cells cultivated in xylose medium. It was found that deletion of *CAT8* led to moderate increase in specific activities of most of the analyzed enzymes involved in xylose metabolism and alcoholic fermentation: xylose reductase, xylulokinase, transketolase, pyruvate decarboxylase and alcohol dehydrogenase. Activity of fructose-1,6-bisphosphatase in *cat8*Δ mutants was slightly increased whereas xylitol dehydrogenase activity was, inversely, decreased as compared to that of the parental strains (Table 2). Cat8 protein is apparently involved in the regulation of the corresponding gene expression. To test this hypothesis, transcription profiles of several potentially involved genes were studied using quantitative reverse-transcription PCR (qRT-PCR). It was found that *cat8*Δ mutant isolated from the wild-type strain cultivated in xylose medium showed higher level of *XYL3, DAS1* and *RPE1* mRNAs whereas expression of the other analyzed genes (*XYL1, XYL2*, *PDC1, TKL1, TAL1, TAL2, FBP1, PCK1*) was quite similar as compared to that of the wild-type strain (Table 3). Strain BEP *cat8*Δ revealed increased expression of *RPE1*, decreased expression of *XYL1, XYL2* and *DAS1* while the expression of other tested genes possessed minor fluctuations relative to that of BEP strain on xylose containing medium (Table 3). Expression of *RPE1* was increased for both deletion mutants to infer this gene as a promising target for overexpression, aiming to increase performance of xylose alcoholic fermentation. We also assayed the relative expression of the studied genes between *O. polymorpha* wild-type strain NCYC495 and the BEP strain as it was not done previously [16]. It showed a substantial enhancement of the expression of genes involved in xylose metabolism in ethanol overproducing strain with especially high increase in expression of *RPE1* gene (Table 3). We speculate that this was achieved by metabolic engineering of the first steps of xylose metabolism but also possibly as a result of classical selection [16].

**Table 1:** Respiration activity of analyzed *O. polymorpha* strains

Strain	Respiration (nanomoles of O_2_ consumed per minute per mg of cells at 30 °C)	
	Glucose as substrate	Xylose as substrate
WT	11.81 ± 0.52	11.35 ± 0.56
*cat8*Δ	8.49 ± 0.07	7.08 ± 0.14
BEP	11.87 ± 0.59	17.17 ± 0.86
BEP *cat8*Δ	13.98 ± 0.70	10.53 ± 0.02

Determinations were performed in distilled air-saturated water with the concentration of cells 0.5 g/L of dry weight and started by addition of 1% carbon substrate (glucose or xylose). The respiratory rate was expressed as nanomoles of O_2_ consumed per minute per mg of cells (dry weight)

**Table 2:** Specific activities of XR (xylose reductase), XDH (xylitol dehydrogenase), XK (xylulokinase), ADH (alcohol dehydrogenase), PDC (pyruvate decarboxylase), FBP (fructose-1,6-bisphosphatase), and TKL (transketolase) in the cells of analyzed *O. polymorpha* strains from third day of xylose alcoholic fermentation at 45 °C

Strain	Activity U/mg of protein						
	XR	XDH	XK	PDC	ADH	TKL	FBP
WT	0.012 ± 0.001	0.011 ± 0.001	–	0.165 ± 0.008	0.103 ± 0.005	0.005 ± 0.002	0.012 ± 0.001
*cat8*Δ	0.014 ± 0.002	0.006 ± 0.001	–	0.183 ± 0.012	0.119 ± 0.001	0.008 ± 0.001	0.014 ± 0.003
BEP	0.023 ± 0.003	0.335 ± 0.004	0.494 ± 0.031	0.323 ± 0.018	0.119 ± 0.020	0.012 ± 0.004	0.011 ± 0.001
BEP *cat8*Δ	0.028 ± 0.002	0.255 ± 0.018	0.629 ± 0.038	0.346 ± 0.006	0.189 ± 0.015	0.019 ± 0.003	0.015 ± 0.002

– Not determined

**Table 3:** The relative expression levels of the particular genes in the parental strains and *cat8*Δ mutants at the third day of xylose alcoholic fermentation at 45 °C

ΔΔCt	Genes										
	*XYL1*	*XYL2*	*XYL3*	*PDC1*	*TKL1*	*DAS1*	*TAL1*	*TAL2*	*RPE1*	*FBP1*	*PCK1*
*cat8*Δ/WT	1.13 ± 0.300	1.05 ± 0.577	2.82 ± 0.438	0.64 ± 0.400	1.10 ± 0.361	2.39 ± 0.342	1.23 ± 0.360	1.21 ± 0.193	2.60 ± 0.486	0.67 ± 0.165	1.10 ± 0.435
BEP *cat8*Δ/BEP	0.36 ± 0.300	0.62 ± 0.085	1.07 ± 0.086	0.88 ± 0.479	0.79 ± 0.175	0.57 ± 0.195	1.11 ± 0.091	1.12 ± 0.067	1.54 ± 0.052	0.74 ± 0.392	0.76 ± 0.140
BEP/WT	7.66 ± 0.971	18.03 ± 0.045	2.76 ± 0.158	1.96 ± 0.380	1.71 ± 0.178	1.70 ± 0.670	9.29 ± 0.138	3.59 ± 0.138	47.60 ± 0.301	1.02 ± 0.274	0.19 ± 0.414

The mRNA quantification was normalized to *ACT1* mRNA

Genes encode: *XYL1*, xylose reductase; *XYL2*, xylitol dehydrogenase; *XYL3*, xylulokinase; *PDC1*, pyruvate decarboxylase; *TKL1*, transketolase; *DAS1*, dihydroxyacetone phosphate synthase or peroxisomal transketolase; *TAL1*, transaldolase; *TAL2*, peroxisomal transaldolase; *RPE1*, ribulosephosphate epimerase; *FBP1*, fructose-1,6-bisphosphatase; *PCK1*, phosphoenolpyruvate carboxykinase

### Ethanol production by mutants with deletion and overexpression of *CAT8* gene in xylose and glucose media

**Fig. 2:**
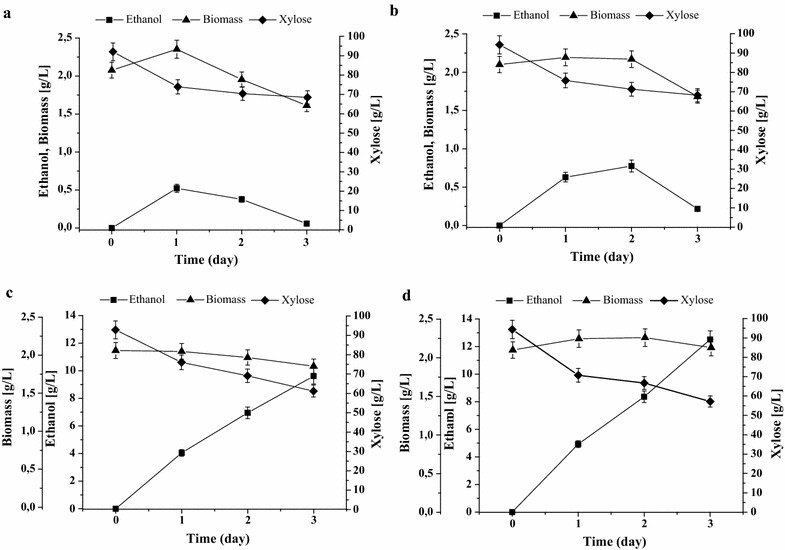
The ethanol production, xylose consumption and biomass accumulation during xylose fermentation at 45 °C of *O. polymorpha* strains: **a** WT, **b** *cat8*∆, **c** BEP, **d** BEP *cat8*∆

**Fig. 3:**
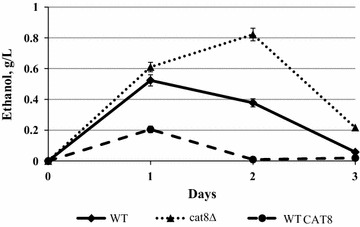
The ethanol production of *O. polymorpha* strains: WT, *cat8*∆ and strain with overexpression of *CAT8* gene (CAT8*) during xylose alcoholic fermentation at 45 °C

Xylose and glucose fermentation of the isolated *cat8*Δ and BEP *cat8*Δ strains was studied under semi-anaerobic conditions (see “Methods” section). It was found that defects of *CAT8* gene leads to 1.5-fold increase in ethanol accumulation on the background of the wild-type strain though concentration of the accumulated ethanol was quite low (Table 4; Fig. 2). At the same time, overexpression of *CAT8* led to decrease in ethanol production from xylose (Fig. 3). Effect of *CAT8* overexpression on glucose fermentation was insignificant (Additional file 4).

**Table 4:** Main parameters of xylose fermentation at 45 °C by the *O. polymorpha* strains tested

Strain	Ethanol (g/L)	Ethanol yield (g/g consumed xylose)	Rate of ethanol synthesis (g/g biomass/h)	Productivity of ethanol synthesis (g/L/h)
WT^a^	0.523 ± 0.054	0.029 ± 0.010	0.009 ± 0.001	0.022 ± 0.001
*cat8*Δ^b^	0.780 ± 0.083	0.034 ± 0.002	0.012 ± 0.001	0.026 ± 0.001
BEP^c^	9.620 ± 0.102	0.300 ± 0.011	0.082 ± 0.002	0.169 ± 0.007
BEP *cat8*Δ^c^	12.51 ± 0.134	0.340 ± 0.015	0.091 ± 0.003	0.205 ± 0.009

^a^ Data of ethanol yield and ethanol (g/L) are represented on YNB medium supplemented with 9% of xylose on the first day (24 h) of fermentation

^b^ 48 h of fermentation

^c^ 72 h of fermentation

It is important to note that deletion of *CAT8* in BEP strain also had a positive effect on ethanol accumulation, which increased by 30% and reached 12.5 g ethanol/L. Increased ethanol production from xylose was accompanied by activated xylose consumption from the medium (Fig. 2). Data of Table 4 show that the strain BEP *cat8*Δ possessed increase in ethanol yield and productivity in xylose medium relative to the parental overproducing strain BEP for 13 and 21%, respectively. Strain BEP *cat8*Δ did not accumulate xylitol (data not shown) similar to that of the parental strain BEP [16]. Thus, we conclude that Cat8 transcription factor is involved in the control of xylose alcoholic fermentation and the deficiency of this protein activates ethanol production from xylose. In contrast, *CAT8* deletion did not have pronounced effect on ethanol production during glucose fermentation both in the wild-type and the BEP strains (Additional file 4). Deletion of *CAT8* gene on both wild-type and BEP backgrounds also did not have effects on alcoholic fermentation of sucrose (data not shown).

## Discussion

The natural xylose-utilizing thermotolerant yeast *O. polymorpha* ferments xylose and glucose at highest temperatures known for yeasts, i.e. at 50 °C [6, 8]. The current work introduces *CAT8* as a gene involved in the regulation of xylose metabolism and alcoholic fermentation in this organism. Prior to this study, the role of *CAT8* in xylose alcoholic fermentation had not been addressed. It was shown that the deletion of this gene in *S. cerevisiae* slightly activated glucose alcoholic fermentation [30]. In contrast, strong activation was observed in *P. guilliermondii* [22] though maximally achieved level of ethanol in the latter species was still very low. In *O. polymorpha CAT8* deletion did not lead to any significant changes in ethanol production from glucose, while a considerable increase in xylose alcoholic fermentation was observed. The reasons for this difference remain to be elucidated; quite possibly the enzymes involved in ethanol production are not activated in *cat8*Δ mutants during glucose fermentation. It has also to be pointed out that cell respiration of *cat8*Δ mutants on xylose was impaired in much higher extent relative to that on glucose as a substrate where BEP *cat8*Δ showed some increase in glucose respiration (Table 1), assuming xylose redirection from the Krebs cycle and oxidative phosphorylation towards ethanol production. The reason of the increase of ethanol production from xylose by *cat8*Δ strains could be explained by activation of xylulokinase, alcohol dehydrogenase and ribulosephosphate epimerase (Tables 2, 3) which could be the limiting factors during xylose alcoholic fermentation.

We observed impaired ethanol and glycerol utilization in *cat8*Δ mutants, suggesting the involvement of *CAT8* in regulation of gluconeogenesis in *O. polymorpha*, similar to that in *S. cerevisiae*. Remarkably, growth on xylose of *cat8*Δ mutant isolated from the wild-type strain was also partially impaired which suggests that xylose can be considered, at least partially, as gluconeogenic substrate. i.e., if hexoses are to some extent synthesized from xylose in gluconeogenesis (from glyceraldehyde-3-phosphate which is synthesized in pentose phosphate pathway), this, together with defects in respiration, especially strong on xylose, could cause the redirection of xylose flux of *cat8*Δ mutants to catabolism and thus the redirection of xylose metabolism to the fermentation mode. Enhanced ethanol production from xylose by *cat8*Δ mutants could also be explained by the observed increase in enzyme activities and transcriptions of genes involved in xylose utilization and alcoholic fermentation (Tables 2, 3). Contrary, the slight increase in specific activity of fructose-1,6-bisphosphatase in *cat8*Δ mutants was observed suggesting differences in Cat8 action between *S. cerevisiae* and *O. polymorpha*. We suggest that growth impairments of *O. polymorpha cat8*Δ mutants on glycerol and ethanol are determined by partial defects in respiration which is critical for growth on gluconeogenic substrates. Quite possible that this is also the reason of xylose growth retardation of *cat8*Δ mutant isolated from the wild-type strain. In spite activity of xylitol dehydrogenase is lowered in *cat8*Δ mutants, it is unlikely that Xyl2 is the limiting enzyme during growth on xylose as our earlier studies showed that deletion of the main paralog *XYL2* (assayed in current manuscript) did not impair growth on xylose at all and deletion of two paralogs of *XYL2* impaired growth still not completely [13].

It is interesting to note that overexpression of *CAT8* has opposite effect on xylose alcoholic fermentation as compared to that in *cat8*Δ mutants as transformants overexpressing *CAT8* gene were characterized by decrease in ethanol production from xylose (Fig. 3). Apparently high amounts of Cat8 activate xylose gluconeogenesis and respiration while inhibit fermentation of this pentose. Deletion or overexpression of *CAT8* had no effect on glucose fermentation suggesting specific involvement of Cat8 protein in regulation of xylose alcoholic fermentation.

Thus, the *CAT8* gene is one of the first identified genes specifically involved in regulation of xylose alcoholic fermentation in the natural xylose-fermenting yeasts. Inactivation of this gene (its knock out) increased ethanol production on backgrounds of the wild-type strain and of the advanced ethanol producer from xylose (BEP). The best ethanol producer from xylose described here, accumulated 30% more ethanol relative to the BEP strain from xylose reported previously and 20–25 times more compared to the wild-type strain [16]. The yield and productivity of ethanol synthesis in BEP *cat8*Δ strain, constructed in this work, for 13 and 21% exceeds those in the reported *O. polymorpha* ethanol overproducer from xylose. Ethanol yield in the BEP *cat8*Δ strain (0.34 g/g xylose) is close to that described for *S. stipitis* (0.35–0.44 g/g xylose) [31] and *S. passalidarum* (0.42 g/g xylose) [32], however, it was achieved for *O. polymorpha* at 45 °C whereas the compared organisms are mesophilic and thus cannot grow and ferment at so high temperature. Among thermotolerant ethanol producing strains the promising one is engineered *K. marxianus* strain with ethanol yield 0.38 g/g xylose at 42 °C, but lower yield at 45 °C (0.27 g/g xylose) [33]. In contrast to recombinant *K. marxianus* strain [33], BEP *cat8*Δ did not accumulate byproduct xylitol at all. Still, the level of increase in ethanol synthesis achieved in this work is not enough for feasible ethanol production from xylose. However, we suggest that the described approach could be useful, in combination with other ones, for future construction of the efficient thermotolerant ethanol producers from xylose.

One may assume that *cat8*Δ mutants of xylose-utilizing recombinant *S. cerevisiae* could be also characterized by an increase in ethanol production from this pentose. It would also be interesting to check the effects of *CAT8* deletion on xylose alcoholic fermentation in the species of natural xylose fermenting yeasts, such as *S. stipitis, S. passalidarum* and others. We hypothesize that the deletion of *CAT8* gene could become a standard approach for development of effective xylose fermenting strains. It would also be of interest to check the role of transcription factors Adr1 [30], and Znf1 [34], Rds2, Sip4 and others [20], in xylose alcoholic fermentation in *O. polymorpha* and other yeast species. Recently, we checked the effects of the knock-out of two *O. polymorpha* homologs of transcriptional regulator *HAP4*, *HAP4*-*A* and *HAP4*-*B*, on xylose growth and fermentation and found only a slight increase in ethanol production from xylose in *hap4*-*A*Δ mutant [35].

We envisage that there are new efficient strategies for additional increase in ethanol production from xylose in *O. polymorpha*. They include autophagy initiation gene *ATG13* [17; Dmytruk, Sibirny, in preparation] and several genes coding for peroxisomal proteins [17; Kurylenko, Ruchala, Vasylyshyn, Dmytruk, Sibirny, in preparation]. Change of expression of the mentioned genes leads to significant and specific increase in ethanol yield from xylose on the background of the wild-type strain. We hope that the manipulation with these gene expression could also be useful for further increase of ethanol production in the described here ethanol overproducer from xylose. Currently our attention is focused to the fermentation of lignocellulosic hydrolysates by constructed xylose fermenting strains. This could constitute an important step towards the establishment of *O. polymorpha* as a promising high-temperature ethanol producer from xylose and other lignocellulosic sugars.

## Conclusions

The mutants of the methylotrophic yeast *Ogataea (Hansenula) polymorpha* with knock out and overexpression of the ortholog of *CAT8* gene coding for transcriptional activator, have been constructed. The *cat8*Δ mutants showed 30–50% increase in ethanol synthesis from xylose. No effect of *CAT8* knock out on ethanol production from glucose was observed. The best strain accumulated 12.5 g of ethanol/L from xylose at 45 °C. Inversely, overexpression of *CAT8* resulted in decrease of ethanol production from this pentose.

## Methods

### Strains, vectors, cultivation condition

The following strains of *O. polymorpha* were used: NCYC495 *leu1*-*1* (wild-type strain), 2EtOH/XYL1m/XYL2/XYL3/BrPA (designated as BEP from best ethanol producer) which is advanced ethanol producer from xylose isolated by combination of the methods of metabolic engineering and classical selection [16]. Yeast cells were grown on YPD (10 g/L yeast extract, 10 g/L peptone, 20 g/L glucose) or mineral medium (6.7 g/L YNB without amino acids, 20 g/L of carbon source—glucose, xylose, glycerol, ethanol) at 37 °C. For the NCYC495 *leu1*-*1* strain, leucine (40 mg/L) was added to the medium. For the selection of yeast transformants on YPD 0.1 g/L of nourseothricin or 0.35 g/L of hygromycin were added. Alcoholic fermentation of yeast strains was fulfilled by cultivation in liquid mineral medium at oxygen-limited conditions at 37 and 45 °C. The conditions were provided by agitation at 140 rpm. 9% xylose or 9% glucose was added into the medium used for the fermentation. The cells were pregrown in 100 mL of liquid YPX medium (1% yeast extract, 2% peptone and 4% xylose) in 300 mL Erlenmeyer flasks at 220 rpm till the mid-exponential growth phase. Than the cells were precipitated by centrifugation, washed by water and inoculated into 40 mL of the fermentation medium in 100 mL Erlenmeyer flasks covered with cotton plugs. The initial biomass concentration for fermentation experiments was 2 g (dry weight)/L. Fermentations were repeated at least in three independent experiments, each performed in triplicate to ensure the results are reproducible. The bars in the figures indicate the ranges of the standard deviation.

The *E. coli* DH5α strain (Φ80d*lacZ*ΔM15, *recA*1, *endA*1, *gyrA*96, *thi*-1, *hsdR*17(r_K_^−^, m_K_^+^), *supE*44, *relA*1, *deoR*, Δ(*lacZYA*-*argF*)U169) was used as a host for plasmid propagation. Strain DH5α was grown at 37 °C in LB medium as described previously [36]. Transformed *E. coli* cells were maintained on a medium containing 100 mg/L of ampicillin.

### Molecular-biology techniques

Standard cloning techniques were carried out as described [36]. Genomic DNA of *O. polymorpha* was isolated using the Wizard^®^ Genomic DNA Purification Kit (Promega, Madison, WI, USA). Restriction endonucleases and DNA ligase (Fermentas, Vilnius, Lithuania) were used according to the manufacturer specifications. Plasmid isolation from *E. coli* was performed with the Wizard^®^ *Plus* SV Minipreps DNA Purification System (Promega, Madison, WI, USA). DNA fragments were separated on a 0.8% agarose (Fisher Scientific, Fair Lawn, NJ, USA) gel. Isolation of fragments from the gel was carried out with a DNA Gel Extraction Kit (Millipore, Bedford, MA, USA). PCR-amplification of the fragments of interest was done with Platinum^®^ *Taq* DNA Polymerase High Fidelity (Invitrogen, Carlsbad, CA, USA) according to the manufacturer specification. PCRs were performed in GeneAmp^®^ PCR System 9700 thermocycler (Applied Biosystems, Foster City, CA, USA). Transformation of the yeast *O. polymorpha* was carried out as described previously [37].

### Construction and analysis of *cat8*Δ* O. polymorpha* deletion mutants

Genomic DNA of *O. polymorpha* NCYC495 *leu 1*-*1* strain was used as template for isolation of 5′ and 3′ uncoding regions of *CAT8* gene by PCR amplifications using primers 5′CAT8 FW/5′CAT8 RW and 3′CAT8 FW/3′CAT8 RW (Sequences of all primers represented in Additional file 5). The resulted 5′CAT8 (671 bp) and 3′CAT8 (697 bp) fragments were EcoRI/BglII or BglII/PstI digested and cloned into EcoRI/PstI linearized vector pUC57. The resulted recombinant was named pUC57-CAT8. Gene *natNT2* (1318 bp) conferring resistance to nourseothricin was amplified using vector pRS41N [38] as a template and primers OK19 and OK20. Obtained fragment was BglII-digested and subcloned into BglII-linearized plasmid pUC57-CAT8. As a result of further genetic manipulations recombinant plasmid pUC57-ΔCAT8-natNT2 was constructed (Additional file 1A). After that, plasmid pUC57-ΔCAT8-natNT2 was NdeI-linearized and transformed into *O. polymorpha* NCYC495 *leu1*-*1* recipient strain using electroporation method. Transformants were selected on the solid YPD medium supplemented with 0.1 g/L of nourseothricin after three days of incubation at 37 °C. Obtained transformants were examined by PCR using genomic DNA of recombinant strains as a template. Transformants with confirmed deletion of *CAT8* were stabilized by altering cultivation in nonselective and selective media and once again examined by PCR. Fragments with predicted size were amplified using pairs of primers homologous to the sequence of selective marker and regions outside from the fragments used for recombination (JR_CAT8_FW/OK20 and OK19/JR_CAT8_RW) (Additional file 1C).

Deletion cassette for isolation of *cat8*Δ mutant on the background of strain BEP was constructed as follows. Genomic DNA of *O. polymorpha* NCYC495 *leu1*-*1* strain was used as template for isolation of 5′ and 3′ uncoding regions of *CAT8* gene by PCR amplifications using primers 5′C8_FW/5′C8_RW and 3′C8_FW/3′C8_RW. The resulted 5′CAT8 (878 bp) and 3′CAT8 (780 bp) fragments were EcoRI/BglII and BglII/PstI double-digested and cloned into EcoRI/PstI linearized vector pUC57. The resulted recombinant was named pUC57-C8. Gene *hphNT1* (1777 bp) conferring resistance to hygromycin was amplified from plasmid pRS42H [38] as a template and primers Hyg_FW and Hyg_RW. Obtained fragment was BglII-digested and subcloned into BglII-linearized plasmid pUC57-C8. Resulted plasmid was designated as pUC57-ΔCAT8-hphNT1 (Additional file 1B). Plasmid pUC57-ΔCAT8-hphNT1 was XbaI-linearized and transformed into BEP strain by electroporation. Transformants were selected on the solid YPD medium supplemented with 0.35 g/L of hygromycin after four day of incubation at 37 °C. Homologous recombination of the deletion cassette with target site was verified by PCR applying the same approach as that described above using pairs of primers JR_CAT8_FW/Hyg RW and Hyg FW/JR_CAT8_RW (Additional file 1D).

### Construction and analysis of *O. polymorpha* strains with overexpression of *CAT8* gene

Plasmid puc19-GAPp-GAPt-natNT2 [39] was used as the basic one for overexpression of *CAT8*. Promoter *GAP* of the gene coding for glyceraldehyde-3-phosphate dehydrogenase was used for *CAT8* overexpression. Genomic DNA of *O. polymorpha* NCYC495 *leu1*-*1* strain was used as template for isolation of *CAT8* gene by PCR amplifications using primers C8_F/C8E_R. After that, gene was XbaI/NotI double-degisted and cloned into XbaI/NotI linearized vector puc19-GAPp-NTC. The resulting plasmid was named p19-GAPp-CAT8-GAPt-natNT2 (Additional file 6). Plasmid p19-GAPp-CAT8-GAPt-natNT2 was ScaI-linearized and transformed into NCYC495 *leu1*-*1* strain by electroporation. Transformants were selected on the solid YPD medium supplemented with 0.1 g/L of nourseothricin after three days of incubation at 37 °C. The transformants were stabilized by cultivation in non-selective media with further shifting to the selective media with nourseothricin. The presence of recombinant *CAT8* gene driven by the *HpGAP* promoter in genomic DNA of stable transformants was confirmed by PCR using primers K_O_644/C8E_R. Overexpression of *CAT8* in the resulted strain was confirmed by qRT-PCR (Additional file 3).

### Respiration activity assay

Cells were grown to the late exponential phase in mineral medium with glucose or xylose, collected, washed in distilled water and starved in mineral medium without carbon source for 16–18 h. Viability of the starved cells was found to be around 70% of that of the non-starved cells by plate count of colony forming units (data not shown). The respiration rate was measured at 30 °C by Yellow Springs Instrument Co. Clark oxygen electrode (model YSI 5300) in a 5 mL reaction vessel. Determinations were performed in distilled air-saturated water with the concentration of cells 0.5 g/L of dry weight from 5 independent cultivations and started by addition of 1% carbon substrate (glucose, xylose). The respiratory rate was expressed as nanomoles of O_2_ consumed per minute per mg of cells (dry weight).

### Biochemical methods

Samples for enzyme activity measurements were taken from the cultures on the third day of xylose fermentation at 45 °C. The enzyme activity was measured directly after the preparation of cell-free extracts. Protein concentration was determined with Folin reagent [40]. The specific activities of XR, XDH and XK in cell extracts were determined spectrophotometrically as described before [14].

TKL activity was assayed spectrophotometrically at 278 nm as previously described with some modifications [41]. In brief, the reaction mixture contained: 50 mM Tris–HCl buffer (pH 7.5), 2.5 mM MgCl_2_, 60 μM TPP, cell extract (0.4 mg of protein). The reaction was started by addition of 100 mM glycol aldehyde.

The PDC activity in cell extracts was determined spectrophotometrically according to the method described earlier [15]. The ADH activity was measured by following the reduction of NAD at 340 nm using 96% ethanol as a substrate as described previously [42]. Briefly, the assay mixture contained 100 mM Tris–HCl (pH 8.0), 2 mM NAD, 100 mM ethanol. The reaction was initiated with the addition of cell extract (0.1 mg of protein).

FBP activity was measured spectrophotometrically in cell extracts as described elsewhere with some modifications [43]. Briefly, the FBP assay was performed in a reaction mixture containing 100 mM Tris–HCl buffer (pH 8.5), 1 mM EDTA, 5 mM MgCl_2_, 2 mM fructose-1,6- diphosphate, 0.4 mM NADP and 1 units of glucose-6-phosphate isomerase and glucose-6-phosphate dehydrogenase. The reaction was initiated with the addition of cell extract (0.4 mg of protein).

All assay experiments were repeated at least twice.

### Quantitative real-time PCR (qRT-PCR)

Expression of the *XYL1*, *XYL2*, *XYL3*, *DAS1*, *TAL2*, *RPE1*, *TAL1*, *PDC1*, *FBP1* and *PCK1* genes was analyzed by real-time PCR. Total RNA was extracted using the GeneMATRIX Universal RNA Purification Kit with DNAse I (EURx Ltd., Gdansk, Poland). RNA was quantified using Picodrop Microliter UV/Vis Spectrophotometer and diluted in RNAse free water. The qRT-PCR was performed by 7500 Fast Real-Time PCR System (The Applied Biosystems, USA) with SG OneStep qRT-PCR kit (EURx Ltd., Gdansk, Poland) using gene-specific primer pairs, RNA as a template and ROX reference passive dye according to the manufacturer’s instructions. The primers pairs used for qRT-PCR are listed in Additional file 5: Table S1. Sequences of tested genes were taken from *O. polymorpha* genome database [28]. In brief, normalized amount of RNA (100 ng) and 0.4 μM of each of the two primers were used in a total reaction volume of 20 μL. The amplification was performed with the following cycling profile: reverse transcription step at 50 °C for 30 min; initial denaturation at 95 °C for 3 min at preparation step; followed by 40 cycles of 15 s at 94 °C and 30 s at 60 °C. Melting curve analysis was performed to verify the specificity and identity of PCR products from 65 to 95 °C in the software of real-time cycler. The amplification for over 35–45 cycles gave abundance of PCR product indicating saturation phase. The fold change of each amplicon in each sample relative to the control sample was normalized to the internal control gene *ACT1* and calculated according to the comparative Ct (ΔΔCt) method. All data points were analyzed in triplicate.

### Analyses

The biomass was determined turbidimetrically with a Helios Gamma spectrophotometer (OD, 590 nm; cuvette, 10 mm) with gravimetric calibration. Concentrations of xylose and ethanol from fermentation in medium broth were analyzed by HPLC (PerkinElmer, Series 2000, USA) with an Aminex HPX-87H ion-exchange column (Bio-Rad, Hercules, USA). A mobile phase of 4 mM H_2_SO_4_ was used at a flow rate 0.6 mL/min and the column temperature was 35 °C. Alternatively, concentrations of ethanol in the medium were determined using alcohol oxidase/peroxidase-based enzymatic kit “Alcotest” [44]. Experiments were performed at least twice.

## Additional files

**Additional file 1.** A, B) Scheme of *CAT8* deletion cassettes (*natNT2*—gene conferring resistance to nourseothricin, *hphNT1*- gene conferring resistance to hygromycin); C) PCR verification of the correct cassette integration into genome of the wild-type strain using primers JR_CAT8 FW/OK20 or OK19/JR_CAT8 RW and genomic DNA of transformants as a template (*cat8*∆—constructed deletion strains; WT*—*recipient strain NCYC495 *leu 1*-*1*; L*—*ladder); D) PCR verification of the correct cassette integration into genome of the BEP strain using primers JR_CAT8 FW/Hyg_RW or Hyg_FW/JR-CAT8 RW and genomic DNA of transformants as a template (BEP *cat8*∆—constructed deletion strains; BEP—recipient strain, L—ladder).

**Additional file 2.** Growth of the mutants with deletion of *CAT8* gene on different carbon sources as compared to the parental strains.

**Additional file 3.** The relative expression levels of the *CAT8* gene in the parental strains and strain with overexpressed *CAT8* gene (*CAT8**) at the third day of xylose alcoholic fermentation at 45 °C. The mRNA quantification was normalized to *ACT1* mRNA.

**Additional file 4.** Ethanol production by parental and recombinant strains of *O. polymorpha*: (A) *cat8*∆ and strain with overexpression of *CAT8* gene (WT *CAT8*); (B) BEP *cat8*∆ during glucose fermentation at 45 °C.

**Additional file 5.** List of primers used in this study (restriction sites are underlined).

**Additional file 6.** Linear scheme of plasmid pUC19-GAPp-CAT8-GAPt-natNT2.

## Abbreviations

BEP: best ethanol producer
SSF: Simultaneous Saccharification and Fermentation
qRT-PCR: quantitative reverse-transcription PCR
XR: xylose reductase
XDH: xylitol dehydrogenase
XK: xylulokinase
TKL: transketolase
ADH: alcohol dehydrogenase
PDC: pyruvate decarboxylase
FBP: fructose-1,6-bisphosphatase
