# Supplementary material for: Transcriptional activator Cat8 is involved in regulation of xylose alcoholic fermentation in the thermotolerant yeast Ogataea (Hansenula) polymorpha
Source: Microb Cell Fact. 2017 Feb 28;16:36. doi: 10.1186/s12934-017-0652-6 (PMC5331723; doi:10.1186/s12934-017-0652-6)
Supplement: Supplementary file 1 — Additional file 1. A, B) Scheme of CAT8 deletion cassettes (natNT2—gene conferring resistance to nourseothricin, hphNT1- gene conferring resistance to hygromycin); C) PCR verification of the correct cassette integration into genome of the wild-type strain using primers JR_CAT8 FW/OK20 or OK19/JR_CAT8 RW and genomic DNA of transformants as a template (cat8∆—constructed deletion strains; WT—recipient strain NCYC495 leu 1-1; L—ladder); D) PCR verification of the correct cassette integration into genome of the BEP strain using primers JR_CAT8 FW/Hyg_RW or Hyg_FW/JR-CAT8 RW and genomic DNA of transformants as a template (BEP cat8∆—constructed deletion strains; BEP—recipient strain, L—ladder). [file 12934_2017_652_MOESM1_ESM.pptx]

## Slide 1
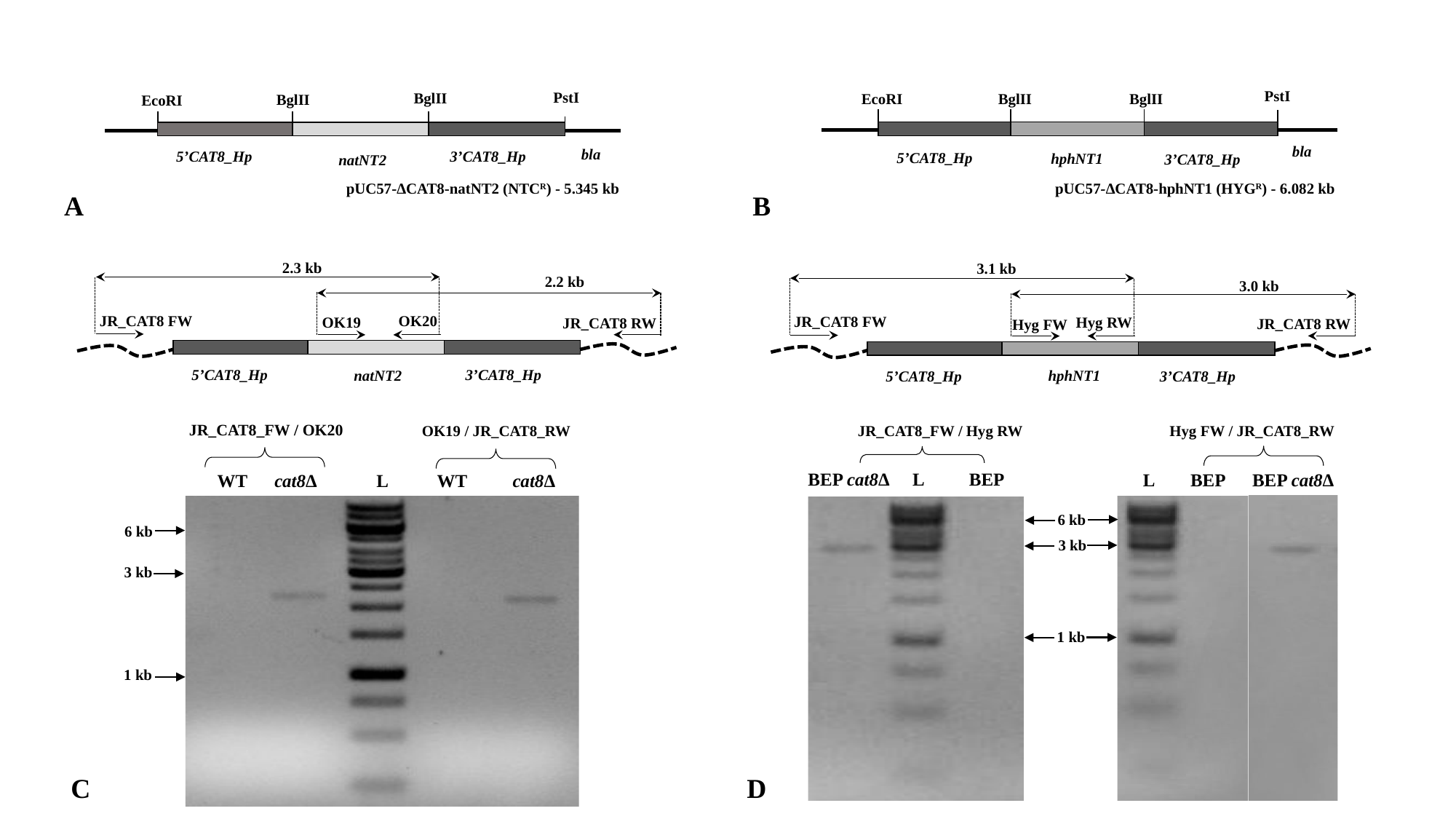

PstI
EcoRI
BglII
BglII
bla
5’CAT8_Hp
3’CAT8_Hp
pUC57-∆CAT8-hphNT1 (HYGR) - 6.082 kb
hphNT1
PstI
BglII
BglII
EcoRI
bla
5’CAT8_Hp
3’CAT8_Hp
natNT2
pUC57-∆CAT8-natNT2 (NTCR) - 5.345 kb
B
A
2.3 kb
2.2 kb
JR_CAT8 FW
OK20
OK19
JR_CAT8 RW
natNT2
5’CAT8_Hp
3’CAT8_Hp
3.1 kb
3.0 kb
JR_CAT8 FW
Hyg RW
JR_CAT8 RW
Hyg FW
hphNT1
5’CAT8_Hp
3’CAT8_Hp
 JR_CAT8_FW / OK20
OK19 / JR_CAT8_RW
Hyg FW / JR_CAT8_RW
 JR_CAT8_FW / Hyg RW
BEP cat8Δ L BEP
 L BEP BEP cat8Δ
 WT cat8Δ L WT cat8Δ
6 kb
6 kb
3 kb
3 kb
1 kb
1 kb
С
D
