# Supplementary material for: Transcriptional activator Cat8 is involved in regulation of xylose alcoholic fermentation in the thermotolerant yeast Ogataea (Hansenula) polymorpha
Source: Microb Cell Fact. 2017 Feb 28;16:36. doi: 10.1186/s12934-017-0652-6 (PMC5331723; doi:10.1186/s12934-017-0652-6)
Supplement: Supplementary file 2 — Additional file 2. Growth of the mutants with deletion of CAT8 gene on different carbon sources as compared to the parental strains. [file 12934_2017_652_MOESM2_ESM.pptx]

## Slide 1
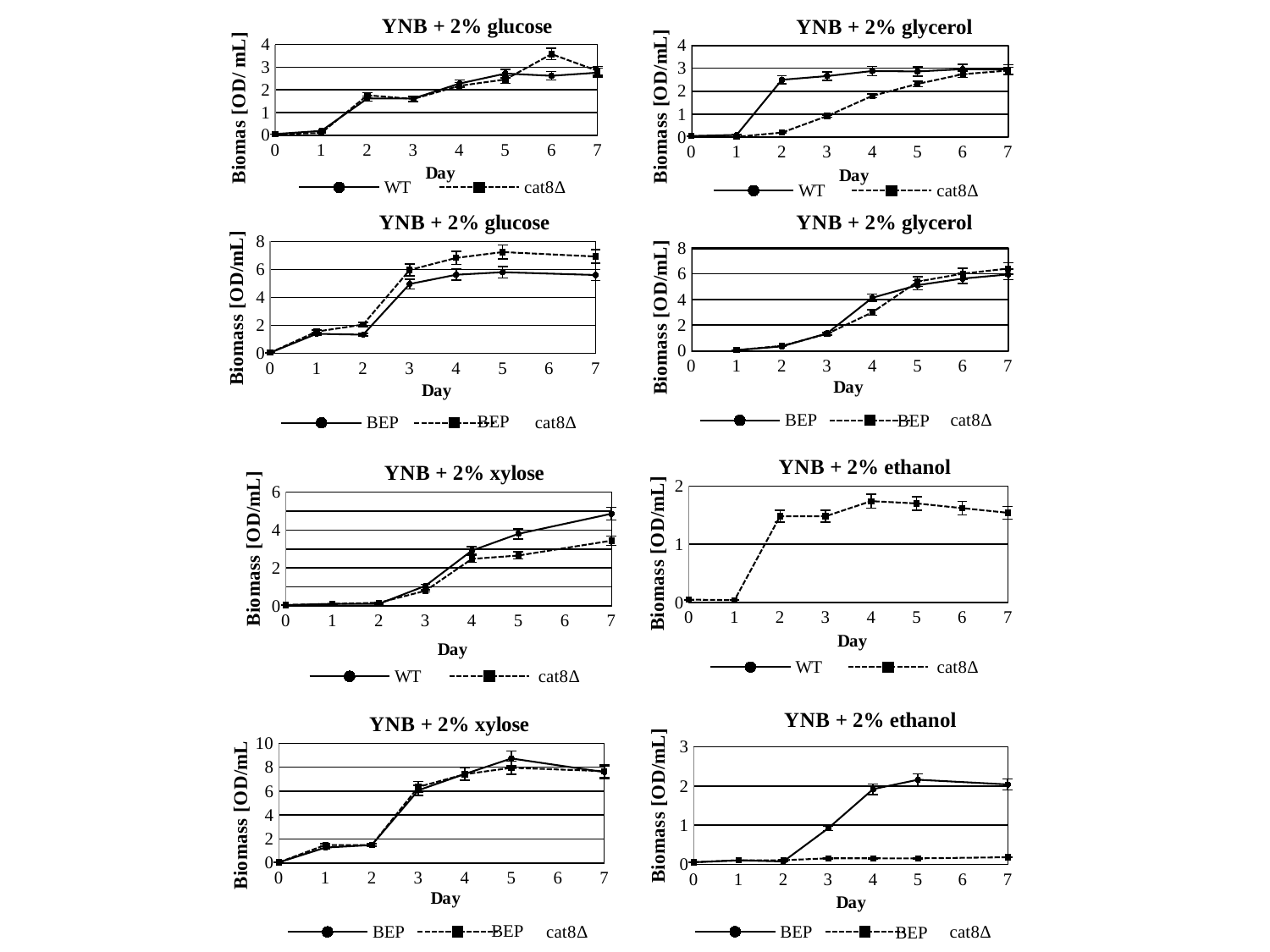

### Chart: YNB + 2% glucose
| Category | WT | cat8Δ |
|---|---|---|
### Chart: YNB + 2% glycerol
| Category | WT | cat8Δ |
|---|---|---|
### Chart: YNB + 2% glucose
| Category | BEP | cat8Δ |
|---|---|---|
### Chart: YNB + 2% ethanol
| Category | WT | cat8Δ |
|---|---|---|
### Chart: YNB + 2% xylose
| Category | WT | cat8Δ |
|---|---|---|
### Chart: YNB + 2% xylose
| Category | BEP | cat8Δ |
|---|---|---|
### Chart: YNB + 2% glycerol
| Category | BEP | cat8Δ |
|---|---|---|BEP
### Chart: YNB + 2% ethanol
| Category | BEP | cat8Δ |
|---|---|---|BEP
BEP
