# Supplementary material for: Transcriptional activator Cat8 is involved in regulation of xylose alcoholic fermentation in the thermotolerant yeast Ogataea (Hansenula) polymorpha
Source: Microb Cell Fact. 2017 Feb 28;16:36. doi: 10.1186/s12934-017-0652-6 (PMC5331723; doi:10.1186/s12934-017-0652-6)
Supplement: Supplementary file 3 — Additional file 3. The relative expression levels of the CAT8 gene in the parental strains and strain with overexpressed CAT8 gene (CAT8*) at the third day of xylose alcoholic fermentation at 45 °C. The mRNA quantification was normalized to ACT1 mRNA. [file 12934_2017_652_MOESM3_ESM.docx]

The relative expression levels of the *CAT8* gene in the parental strains and strain with overexpressed *CAT8* gene (*CAT8**) at the third day of xylose alcoholic fermentation at 45˚C. The mRNA quantification was normalized to *ACT1* mRNA.

| **Strain** | **Relative quantity of *CAT8 Hp*** |
| --- | --- |
| BEP | 1.00 ± 0.160 |
| WT | 1.00 ± 0.091 |
| WT *CAT8** | 2.65 ± 0.098 |
