# Supplementary figures and images for: Transcriptional activator Cat8 is involved in regulation of xylose alcoholic fermentation in the thermotolerant yeast Ogataea (Hansenula) polymorpha
Source: Microb Cell Fact. 2017 Feb 28;16:36. doi: 10.1186/s12934-017-0652-6 (PMC5331723; doi:10.1186/s12934-017-0652-6)

## Slide 1
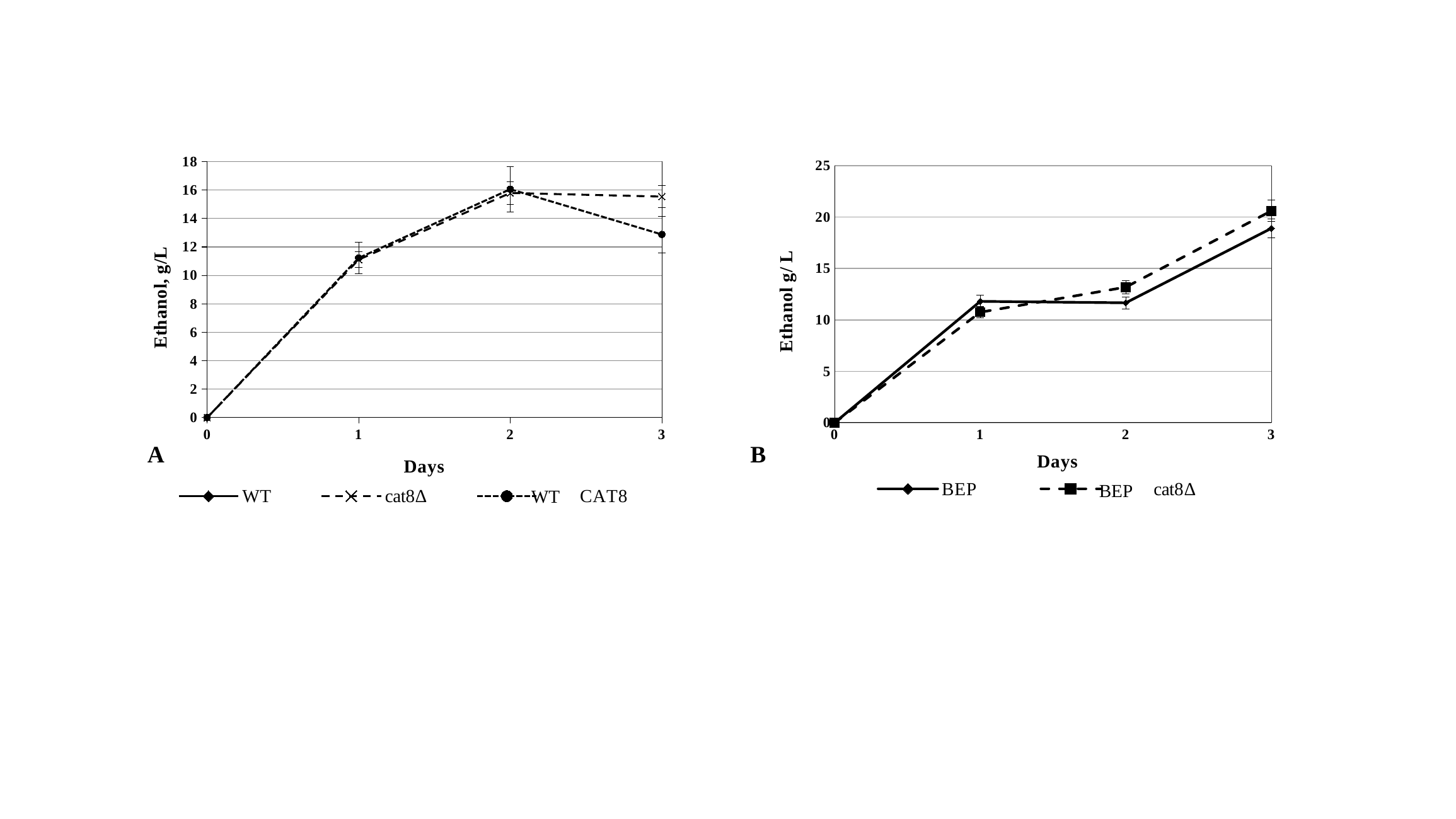

### Chart
| Category | BEP | cat8Δ |
|---|---|---|
### Chart
| Category | WT | cat8∆ | CAT8 |
|---|---|---|---|A
WT
BEP

Supplement: Supplementary file 4 — Additional file 4. Ethanol production by parental and recombinant strains of O. polymorpha: (A) cat8∆ and strain with overexpression of CAT8 gene (WT CAT8); (B) BEP cat8∆ during glucose fermentation at 45 °C. [file 12934_2017_652_MOESM4_ESM.pptx]

## Slide 1
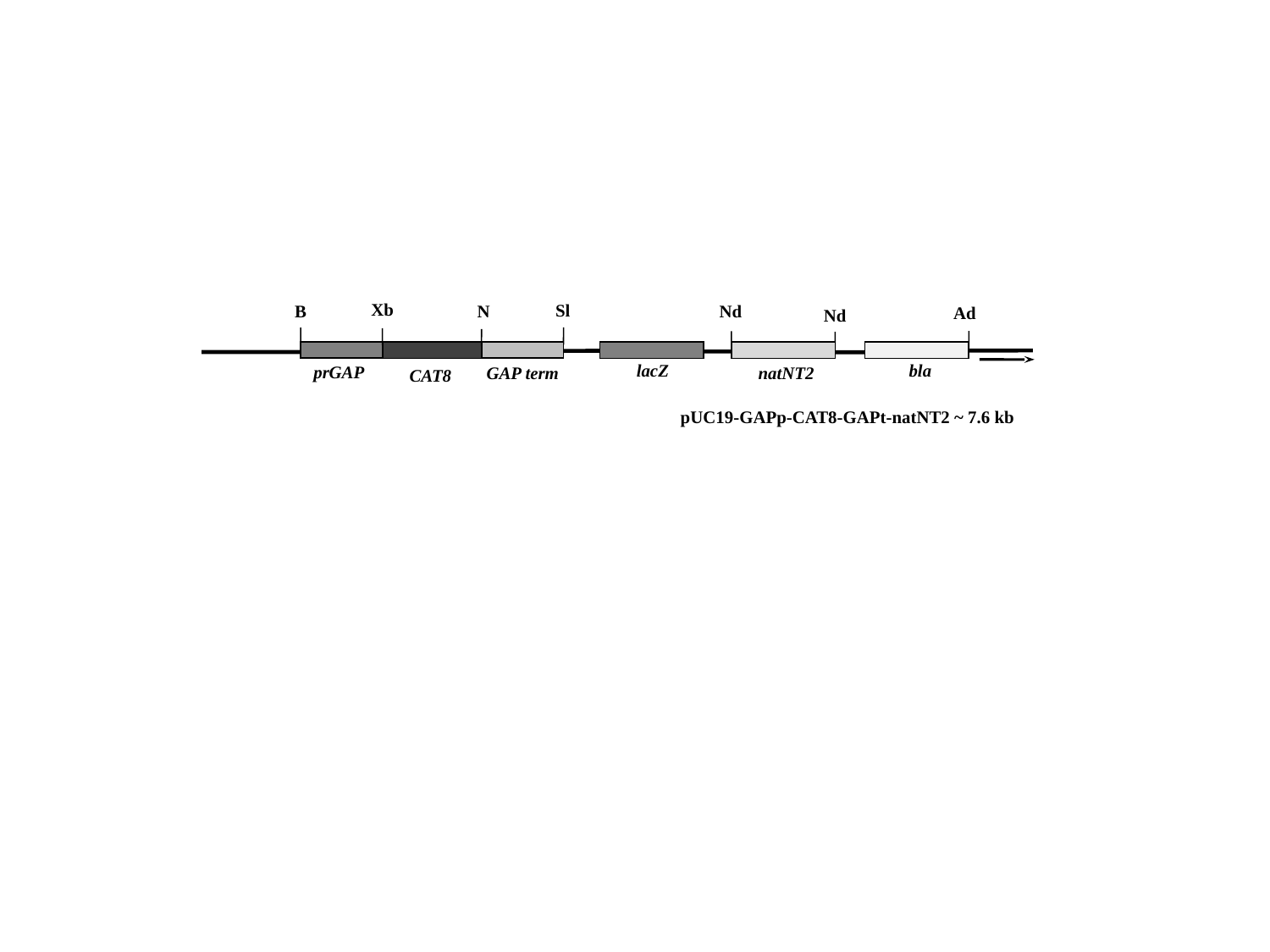

Xb
Sl
N
Nd
B
Ad
Nd
lacZ
bla
GAP term
natNT2
prGAP
CAT8
pUC19-GAPp-CAT8-GAPt-natNT2 ~ 7.6 kb

Supplement: Supplementary file 6 — Additional file 6. Linear scheme of plasmid pUC19-GAPp-CAT8-GAPt-natNT2. [file 12934_2017_652_MOESM6_ESM.pptx]
