# Supplementary material for: Transcriptional activator Cat8 is involved in regulation of xylose alcoholic fermentation in the thermotolerant yeast Ogataea (Hansenula) polymorpha
Source: Microb Cell Fact. 2017 Feb 28;16:36. doi: 10.1186/s12934-017-0652-6 (PMC5331723; doi:10.1186/s12934-017-0652-6)
Supplement: Supplementary file 5 — Additional file 5. List of primers used in this study (restriction sites are underlined). [file 12934_2017_652_MOESM5_ESM.docx]

List of primers used in this study (restriction sites are underlined).

| **Primer name** | **Primer sequence 5’-3’** |
| --- | --- |
| 5’CAT8 FW | CCG GAA TTC CAC TTA CTC TCA GTA CTC TAA AG |
| 5’CAT8 RW | CAT AAT ACA CGA GTC AAT TC AGATCT G CGG CAT TCT TGG CTT ATC AG |
| 3’CAT8 FW | CTG ATA AGC CAA GAA TGC CGC AGATCT GAA TTG ACT CGT GTA TTA TG |
| 3’CAT8 RW | AAA CTGCAG GAA GGC CTT CTT GGA GAG AG |
| OK19 | AGATCT ATA ACT TCG TAT AGC ATA CAT TAT ACG AAG TTA TCT TAA CTA TGC GGC ATC |
| OK20 | AGATCT ATA ACT TCG TAT AAT GTA TGC TAT ACG AAG TTA TCC GAG ATT CAT CAA CTC ATT GC |
| 5’C8_FW | CCG GAATTC CTT GTG TTA GTA CAA CTC TCA GTG |
| 5’C8_RW | CTG AGC TGG TCC TGT A AGATCT GT GTA TGT TCC TCT G |
| 3’C8_FW | CAG AGG AAC ATA CAC AGATCT TAC AGG ACC AGC TCA G |
| 3’C8 RW | AAA CTGCAG CTG CAA GGA GCA CGG TAT TGA G |
| Hyg_FW | GGA AGATCT ATA ACT TCG TAT AGC ATA CAT TAT ACG AAG TTA TGT GAT GAC GGT GAA AAC CTC TG |
| Hyg_RW | GGA AGA TCT ATA ACT TCG TAT AAT GTA TGC TAT ACG AAG TTA TCC CAA AAC CTT CTC AAG CAA G |
| C8_F | CGC GCG GCC GCT CAA TTC CAC ATT TGC TC |
| C8E_R | AAA TCT AGA ATG CCG CCT CCA TCG CCG CCA G |
| Ko644 | CGC GGATCC TAGACCACATCCGTGCACCAG |
| XYL1f | CTC ACA AGG TCA CCC CTG |
| XYL1r | GAT CCT GGT TCA AAG AGC TG |
| XYL2f | GCT GAG GTT GCA GAG AGA G |
| XYL2r | TCC TTG CTG AAG TCG TAA GC |
| XYL3f | TGA CTT GAG CGA TGC ATG C |
| XYL3r | CAT TTC CAA GCT TCT CAA CG |
| DAS1f | GAC GCT CGG TTT TGA GAA AG |
| DAS1r | GTA CGC TTC GAC CTT CTT G |
| TAL2f | AGC GAT GGT CAA ATC GAA TAC |
| TAL2r | TAA ACT TTC AGT GTC GCT TGC |
| RPE1f | ATG ATG CCC AAG GTT GAG AC |
| RPE1r | AGT TCT TTC GAC ACG TAA TCT C |
| TAL1f | TGG CAG GAT GTG ACT ACT TG |
| TAL1r | TTT TCA GTG GCC ATG GCA TC |
| PDC1f | cac agt aca aca tga tcc ag |
| PDC1r | ATC TTG GCT TGC TTG ACA AG |
| FBP1f | AGA CGT GCA CAG AAC ACT G |
| FBP1r | GCT TCT CTC GTG GAT CTT C |
| PCK1f | ATT CTG GAT GCC ATC CAC TC |
| PCK1r | GTT CTC CAT AAA CAG CTG TGC |
| ACT1f | GTC ATG TCT GGT GGT ACT AC |
| ACT1r | TTG GAA ATC CAC ATC TGT TGG |
